# Hypothermic Oxygenated Machine Perfusion of Liver Grafts from Brain-Dead Donors

**DOI:** 10.1038/s41598-019-45843-3

**Published:** 2019-06-27

**Authors:** Damiano Patrono, Astrid Surra, Giorgia Catalano, Giorgia Rizza, Paola Berchialla, Silvia Martini, Francesco Tandoi, Francesco Lupo, Stefano Mirabella, Chiara Stratta, Mauro Salizzoni, Renato Romagnoli

**Affiliations:** 1 General Surgery 2U - Liver Transplant Unit, A.O.U. Città della Salute e della Scienza di Torino, University of Turin, Turin, Italy; 2 Department of Clinical and Biological Sciences, University of Turin, Turin, Italy; 3 Gastrohepatology Unit, A.O.U. Città della Salute e della Scienza di Torino, Turin, Italy; 4 Anesthesia Department 2, A.O.U. Città della Salute e della Scienza di Torino, Turin, Italy

**Keywords:** Medical research, Health care

## Abstract

Hypothermic oxygenated machine perfusion (HOPE) was introduced in liver transplantation (LT) to mitigate ischemia-reperfusion injury. Available clinical data mainly concern LT with donors after circulatory-determined death, whereas data on brain-dead donors (DBD) are scarce. To assess the impact of end-ischemic HOPE in DBD LT, data on primary adult LTs performed between March 2016 and June 2018 were analyzed. HOPE was used in selected cases of donor age >80 years, apparent severe graft steatosis, or ischemia time ≥10 hours. Outcomes of HOPE-treated cases were compared with those after static cold storage. Propensity score matching (1:2) and Bayesian model averaging were used to overcome selection bias. During the study period, 25 (8.5%) out of 294 grafts were treated with HOPE. After matching, HOPE was associated with a lower severe post-reperfusion syndrome (PRS) rate (4% versus 20%, p = 0.13) and stage 2–3 acute kidney injury (AKI) (16% versus 42%, p = 0.046). Furthermore, Bayesian model averaging showed lower transaminases peak and a lower early allograft dysfunction (EAD) rate after HOPE. A steeper decline in arterial graft resistance throughout perfusion was associated with lower EAD rate. HOPE determines a significant reduction of ischemia reperfusion injury in DBD LT.

## Introduction

The unmet gap between organ demand and supply in recent years has pushed the liver transplant (LT) community to make increasing use of so-called extended criteria or marginal donors (ECD). Although criteria to define marginality are arbitrary, extended criteria grafts are most frequently those with moderate or severe graft steatosis or from donors of an advanced age or after circulatory death (DCD). Utilizing these grafts has been associated with an increased rate of primary non-function (PNF), early allograft dysfunction (EAD), graft cholangiopathy (GC), and with reduced graft survival^1–6^. In this regard, various machine perfusion techniques have emerged as valuable alternatives to static cold storage (SCS), allowing a safer utilization of ECDs^7–15^.

The term ‘machine perfusion’ covers a variety of techniques characterized by different settings, timing and technology. The potential benefits and indications of each of these factors are still under investigation^7,11–14,16–18^. Amongst these, end-ischemic hypothermic oxygenated machine perfusion (HOPE) has been associated with improved post-transplant outcomes, especially with grafts from DCD donors. Although use of machine perfusion may also be beneficial for grafts from donors after brain death (DBD)^19^, clinical data in this specific area are very limited.

**Figure 1:**
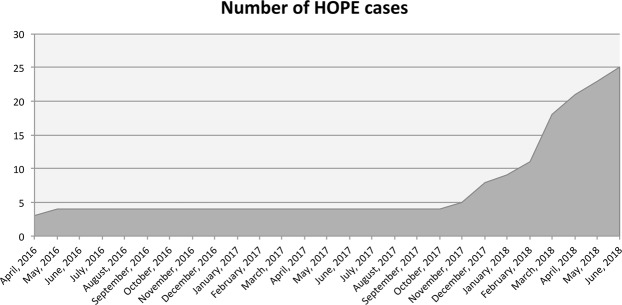
Graphic outlining cumulative number of HOPE cases performed throughout study period.

With a view to improve LT outcomes with grafts from DBD, we implemented a program of dual (i.e. simultaneous perfusion of both portal vein and hepatic artery) HOPE at our center, which began in April 2016. After an initial favorable experience^20^, HOPE was employed in selected cases of DBD liver transplantation following an established protocol since November 2017 (Fig. 1). This article reports our pilot experience with the first 25 cases of HOPE at our Center.

## Patients and Methods

### Study design

Data on all consecutive primary adult (>18-year-old) DBD LTs performed between March 16^th^, 2016 and June 12^th^, 2018, including baseline patient and recipient features, prognostic scores^21–23^, transplant operation details, preservation time, use of HOPE, and postoperative outcomes, were prospectively collected and retrospectively analyzed. Retransplants and DCD LTs were excluded from the analysis. Minimal follow-up was six months. At the entry on the waiting list, all patients signed a consent form agreeing to the possibility of receiving a graft treated with preservation techniques other than static cold storage, including machine perfusion. Due to the retrospective observational design, no specific approval was sought from the local Institutional Review Board; by Italian law (n° 91/1999), regional transplant centers are custodians of recipient biomedical data, including for research purposes.

After the first four cases performed in April-May 2016, machine perfusion use was considered from November 2017 in the following scenarios: (1) donor age ≥80 years; (2) donor body mass index (BMI) ≥30 kg/m^2^ associated with apparent severe graft steatosis as assessed by the retrieval surgeon; (3) logistic issues determining expected ischemia time ≥10 hours. As our center practice does not include pre-transplant routine histologic assessment of the graft by liver biopsy and frozen section examination, graft macroscopic appearance and donor BMI were used as surrogates of graft steatosis. Assessment of graft macro- and microsteatosis was carried out on the biopsy obtained after graft reperfusion into the recipient.

Endpoints included: (1) peak levels of aspartate aminotransferase (AST) and alanine aminotransferase (ALT); (2) lactate level in the recipient at the end of transplant operation; (3) duration of hospital and intensive care unit stay; (4) rate of EAD (as defined by Olthoff *et al*.^24^); (5) moderate-to-severe acute kidney injury (AKI) rate; (6) significant post-reperfusion syndrome (PRS) rate; (7) acute rejection rate; (8) grade ≥3 complications rate (according to Dindo *et al*.^25^); (9) biliary complications rate at 6 months; (10) patient and graft survival. AKI was defined and graded according to 2012 KDIGO guidelines^26^. Severe PRS was defined according to Hilmi *et al*. as a persistent drop in arterial blood pressure (>30% of the anhepatic level), asystole or hemodinamically significant arrhythmias^27^. Biliary complications were classified as anastomotic (including strictures and fistulae) and extra-anastomotic. Non-anastomotic strictures were defined according to Weeder *et al*.^28^ as a combination of narrowing and dilatations (or intraparenchymal leakage) of the larger intra- and extrahepatic donor bile ducts, either with or without intraluminal sludge and cast formation, and in the presence of a patent hepatic artery. As proposed by deVries *et al*.^29^ we used the term’post-transplant cholangiopathy’ (PTC) to identify patients with non-anastomotic strictures. Each type of biliary complication was further classified according to the presence of symptoms and the need for operative treatment.

**Figure 2:**
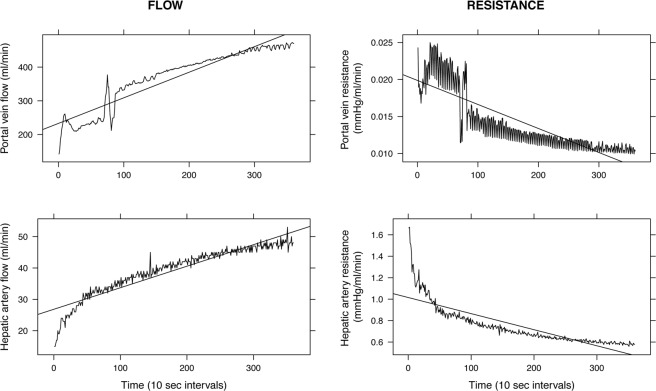
Graphs depicting flow and resistance trend during the first hour of machine perfusion. Similar plots were generated for every case with available data in order to calculate flow and resistance slopes.

In order to evaluate HOPE’s potential in assessing graft quality or viability, we analyzed the association between graft vascular resistances during the first hour of machine perfusion and post-LT AST and ALT peak levels, and EAD onset. For each perfusion, data concerning flow and pressure values measured at 10-second intervals were exported from the perfusion device using the software provided by the manufacturer. We considered flow (ml/min) and resistance (mmHg/ml/min) values obtained from both portal vein and hepatic artery circuits at the end of the first hour of HOPE. For analysis, we considered both absolute values and values normalized to liver graft weight (kg). Finally, for each perfusion circuit, flow and resistance values measured at 10-seconds interval during the first HOPE hour were plotted against time to calculate slopes, intended as mean flow and resistance variation per 10-second interval (Fig. 2).

### Procedural details

After retrieval, liver grafts were stored in cold Celsior^®^ solution (IGL^®^, Lissieu, France) during preservation and backtable preparation, until they were flushed with chilled 5% albumin before implantation in the recipient. The bile duct was accurately flushed at the start of cold perfusion and before packaging with chilled saline. Liver graft was weighed at the end of backtable preparation. In HOPE cases, liver graft was connected to the perfusion device immediately after backtable preparation without being flushed. Livers preserved by static cold storage were flushed with a supplementary 1 L of preservation solution through the hepatic artery at the end of backtable preparation. Standard transplant technique was piggyback cavo-cavostomy with retro-hepatic vena cava preservation, portal reperfusion first, and end-to-end biliary anastomosis with a 2.5 Fr T-tube. In selected cases in which it was not possible to use the piggyback technique, inferior vena cava replacement was carried out; veno-venous bypass was never employed. Biliary reconstruction by hepatico-jejunostomy was selectively used in patients with primary sclerosing cholangitis, or if the recipient bile duct was deemed unsuitable for biliary reconstruction. Immunosuppression was based on tacrolimus (target through level 8–10 ng/ml), mycophenolate mofetil (500 mg b.d. in patients with no leukopenia or thrombocytopenia) and steroids (methylprednisolone 1,000 mg intra-operatively; prednisone 20 mg o.d. from postoperative day 1), which were tapered and stopped 3 months after transplant. Induction with basiliximab was administered to patients with autoimmune or cholestatic liver disease.

In patients with a T-tube, a cholangiogram was routinely obtained 3 months after transplant, after which the T-tube was removed. Other investigations (e.g. magnetic resonance cholangio-pancreatography) were performed where clinically indicated.

### Machine perfusion technique and logistics

End-ischemic HOPE was applied as previously described^7^. Briefly, in HOPE cases, standard graft backtable preparation was initiated upon arrival at our transplant center, while the LiverAssist^®^ device (OrganAssist^®^, Groningen, The Netherlands) was primed with 3 L of Belzer MPS^®^ solution (Bridge to Life Europe Ltd, Wandsworth, London, UK). The coeliac trunk and superior mesenteric vein were subsequently cannulated leaving a side branch open (most commonly gastroduodenal artery and splenic vein) to purge air from the circuit before starting perfusion. When an aberrant hepatic artery was encountered (most frequently an accessory right hepatic artery to be anastomosed to gastroduodenal artery stump), we carried out the appropriate vascular reconstruction at the end of backtable preparation, before machine perfusion. Pressure was set at 5 (continuous) and 25 mmHg (pulsatile) in the portal vein and hepatic artery circuit, respectively; gas analyses were obtained every 30 minutes to check if target oxygen pressure in the perfusate (~600 mmHg) was reached. The reservoir temperature was ~10 °C throughout perfusion. Dual-HOPE was performed in all but two cases, in which only the portal vein was perfused due to a the hepatic artery pump unit malfunction. In these cases, during the priming of the device, we observed a malfunction of the rotary pump with no flow in the hepatic artery unit. The hepatic artery unit was then shut down and HOPE was carried out through portal vein circuit only. Based on the study by Schlegel *et al*.^30^, the start of the transplant operation was coordinated to allow for a minimum HOPE time of 90 minutes. HOPE was stopped at the end of recipient hepatectomy, when the graft was disconnected from the device and transferred to recipient’s table for implantation.

### Statistical analysis

Results are expressed as mean and standard deviation (SD) or as a number and percentage. HOPE cases were first compared with those in which liver was preserved by SCS by using non-parametric Mann-Whitney U test, Chi-squared or Fisher’s exact tests, as appropriate. To overcome the selection bias due to the allocation of HOPE to marginal grafts, propensity score matching with a 1:2 ratio was carried out^31^. Variables used to calculate the propensity score were donor age, macrovesicular steatosis ≥15%, donor BMI, laboratory model for end-stage liver disease (MELD) at LT, donor risk index (DRI) and balance of risk (BAR) score. The matched cohort was built by selecting two patients for every HOPE-treated patient in the control group who had the nearest propensity score. Kaplan-Meier survival estimates were used for survival analysis in the unmatched and matched cohorts. Finally, the association between early outcomes (AST and ALT post-LT peak levels and EAD onset) and the flow and resistance values measured during or at the end of the first hour of machine perfusion was analyzed using linear or logistic regressions as appropriate, and Mann-Whitney U test.

The propensity score analysis does not take account of the uncertainty in the selection of variables in the propensity score equation. Moreover, residual imbalance between treated and untreated subjects may still be present due to the small sample size of the matched cohort. To address these issues, a further analysis using Bayesian Model Averaging (BMA) was carried out on the whole cohort^32^. In short, this approach allows for averaging among all possible statistical models, which are supported by the data, to obtain an unbiased estimate of the effect size of the different variables on the analyzed endpoint^33^. The percentage of inclusion (PI) expresses in which percentage of all possible models the variable has been included. The number of possible models is given by all possible combinations of baseline covariates that have a posterior model probability of at least 1/20. PI can be directly interpreted as the probability of a variable being included in the model and suggests which variables have a confounding effect on the outcome and should be used for adjusting. The effect on the outcome is expressed as an odds ratio (binary outcome) or as a mean variation of the outcome variable per unit variation of the variable in the model (continuous outcome). Finally, the probability of a significant result, i.e. the probability odds ratio is less than 0 or the probability the mean variation is less than 0, is provided as a measure of the size of the association. All analyses were performed with R version 3.3.3.

## Results

End-ischemic HOPE was employed in 25 (8.5%) out of 294 primary adult DBD LTs performed during the study period. Mean (SD) perfusion time was 186 (49) minutes. No adverse event related to machine perfusion was observed. Indications for HOPE were distributed as follows: donor age ≥80 years, n = 12; macroscopic steatotic appearance of the graft at retrieval + donor BMI ≥30 Kg/m^2^, n = 11; logistic issues associated with expected ischemia time ≥10 hours, n = 2. In these last two cases we had to call for a back-up recipient after the scheduled one was deemed unsuitable due to a previously undiagnosed severe pulmonary hypertension discovered during anesthetic preparation in one case, or to extra-hepatic dissemination of hepatocellular carcinoma discovered at laparotomy in the other. Total preservation time was 10 hours and 10 hours 33 minutes, respectively.

As summarized in Table 1, baseline patient features were comparable between the two groups, whereas there was a significant difference in terms of donor age (60.9 [18.4] versus 74.3 [10.9] years; p < 0.001), donor age x recipient MELD (D-MELD) (850 [442] versus 1110 [583]; p = 0.007), DRI (1.78 [0.51] versus 2.09 [0.52]; p = 0.004) and cold ischemia time (393 [82] versus 499 [59] minutes; p < 0.001). To allow for a meaningful comparison between the two groups, a control group was selected using 1:2 propensity score-based matching, allowing good balance between the two groups (Table 1). We deliberately chose not to balance on cold ischemia time, as this difference was due to HOPE logistics. Thus, significant unbalance concerning cold ischemia time persisted in the matched cohort (391 [72] versus 499 [59] minutes; p < 0.001).

**Table 1:** Patient, graft and surgical features in the whole and matched cohorts.

	Whole cohort			Matched cohort		
	SCS (n = 269)	HOPE (n = 25)	p*	SCS (n = 50)	HOPE (n = 25)	p*
**Recipient features**						
Age (y)	55.3 (8.7)	56.3 (9)	0.60	55.9 (7.4)	56.3 (9)	0.85
Sex (male)	200 (74.3%)	15 (60.0%)	0.19	37 (74.0%)	15 (60.0%)	0.33
BMI (Kg/m^2^)	25.2 (3.7)	25 (3.3)	0.74	26.2 (4.6)	25 (3.3)	0.24
MELD	14.3 (7.3)	15.3 (8.6)	0.52	15.5 (8.5)	15.3 (8.6)	0.93
Creatinine at LT (mg/dl)	1.08 (1.05)	0.96 (0.39)	0.59	0.94 (0.46)	0.96 (0.39)	0.79
RRT at LT	13 (4.8%)	1 (4.0%)	1.00	3 (6.0%)	1 (4.0%)	1.00
Previous major abdominal surgery	82 (30.5%)	11 (44.0%)	0.25	13 (26.0%)	11 (44.0%)	0.19
Life support	5 (1.9%)	1 (4.0%)	1.00	1 (2.0%)	1 (4.0%)	1.00
Ascites at LT	99 (36.8%)	8 (32.0%)	0.77	20 (40.0%)	8 (32.0%)	0.67
History of portal vein thrombosis	22 (8.2%)	3 (12.0%)	0.79	4 (8.0%)	3 (12.0%)	0.89
HCC	147 (54.6%)	17 (68.0%)	0.28	28 (56.0%)	17 (68.0%)	0.45
**Donor features**						
Age (y)	60.9 (18.4)	74.3 (10.9)	<0.001	74.9 (10.3)	74.3 (10.9)	0.81
BMI (Kg/m^2^)	25.5 (4.5)	26.7 (3.9)	0.19	26.1 (3.8)	26.7 (3.9)	0.51
Macrosteatosis (%)	4.7 (8.4)	7.4 (10.1)	0.14	6.3 (8.9)	7.4 (10.1)	0.66
Macrosteatosis ≥15%	34 (12.6%)	5 (20.0%)	0.54	10 (20.0%)	5 (20.0%)	1.00
Graft weight (gr)	1527 (364)	1432 (346)	0.21	1431 (290)	1432 (346)	0.99
**Matching and prognostic scores**						
D-MELD	850 (442)	1110 (583)	0.007	1118 (481)	1110 (583)	0.95
BAR	8.7 (7.6)	10 (8)	0.44	9.8 (7.9)	10 (8)	0.94
DRI	1.78 (0.51)	2.09 (0.52)	0.004	2.15 (0.42)	2.09 (0.52)	0.64
GRBWR (%)	2.15 (0.52)	1.95 (0.42)	0.07	1.98 (0.43)	1.95 (0.42)	0.80
**Preservation and operation details**						
Total preservation time (min)	393 (82)	499 (59)	<0.001	391 (72)	499 (59)	<0.001
Cold ischemia time (min)	393 (82)	311 (53)	<0.001	391 (72)	311 (53)	<0.001
Perfusion time (min)	na	186 (49)		na	186 (49)	
Rec. warm ischemia time (min)	24 (8)	23 (7)	0.52	24 (5)	23 (7)	0.65
Packed red blood cells units	7.3 (11.3)	5.2 (5.8)	0.35	8.4 (12.7)	5.2 (5.8)	0.23
Plasma (mL)	2167 (2750)	1680 (1864)	0.386	2317 (2749)	1680 (1864)	0.30
Colloids (mL)	1390 (979)	1756 (3409)	0.20	1428 (949)	1756 (3409)	0.53
Crystalloids (mL)	7242 (3621)	6930 (3724)	0.68	6848 (3127)	6930 (3724)	0.92

Data are expressed as mean (standard deviation) or number (percentage). *p-value referring to Mann-Whitney, Chi-square or Fisher’s test, as appropriate. Abbreviations: SCS, static cold storage; HOPE, hypothermic oxygenated machine perfusion; y, years; BMI, body mass index; MELD, model for end-stage liver disease; LT, liver transplant; RRT, renal replacement therapy; HCC, hepatocellular carcinoma; D-MELD, donor age * MELD; BAR, balance of risk score; DRI, donor risk index score; GRBWR, graft-to-recipient body weight ratio.

### HOPE effect on early LT outcomes

There were no cases of PNF. Outcome analysis in the whole cohort (Table 2) showed a trend towards a reduction of stage 2–3 AKI in the HOPE group (87 [32.3%] versus 4 [16%]; p = 0.140). In the matched cohort, HOPE treatment was associated with a significant reduction of stage 2–3 AKI rate (odds ratio, OR, [95% confidence interval, CI]: 0.29 [0.08–0.86]; p = 0.024) and with a trend towards a reduction of severe PRS (OR [95% CI]: 0.24 [0.02–1.11]; p = 0.069). There was no difference concerning post-LT dialysis requirement in the unmatched (8 [3%] versus 1 [4%]; p = 1) and matched (2 [4%] versus 1 [4%]; p = 1) cohorts. However, the only patient who required dialysis after LT in the HOPE group was already on dialysis pre-transplant. No significant differences were observed concerning AST and ALT peak levels and EAD rate, as well as other analyzed endpoints (Tables 2 and 3).

**Table 2:** Outcomes in the whole and matched cohorts.

	Whole cohort			Matched cohort		
	SCS (n = 269)	HOPE (n = 25)	p*	SCS (n = 50)	HOPE (n = 25)	p*
Stage 2–3 acute kidney injury	87 (32.3%)	4 (16.0%)	0.14	21 (42.0%)	4 (16.0%)	0.046
Dialysis post-LT	8 (3.0%)	1 (4.0%)	1.00	2 (4.0%)	1 (4.0%)	1.00
Severe post-reperfusion syndrome	42 (15.6%)	1 (4.0%)	0.20	10 (20.0%)	1 (4.0%)	0.13
Early allograft dysfunction	86 (32.0%)	8 (32.0%)	1.00	17 (34.0%)	8 (32.0%)	1.00
ALT peak (IU/L)	973 (889)	792 (773)	0.32	817 (540)	792 (773)	0.87
AST peak (IU/L)	1560 (1238)	1425 (1729)	0.62	1498 (1034)	1425 (1729)	0.82
Lactate at the end of LT	2.43 (1.49)	2.46 (1.24)	0.91	2.75 (2.22%)	2.46 (1.24)	0.54
Grade ≥3 complications	54 (20.1%)	5 (20.0%)	1.00	11 (22.0%)	5 (20.0%)	1.00
Acute rejection	23 (8.6%)	4 (16.0%)	0.39	6 (12.0%)	4 (16.0%)	0.90
Hospital stay (days)	15.9 (17.4)	15.1 (9.4)	0.81	14.3 (6.6)	15.1 (9.4)	0.69
ITU stay (days)	4.2 (3.2)	3.9 (4.0)	0.73	4.2 (2.6)	3.9 (4.0)	0.74
Biliary complications	49 (18.2%)	6 (24.0%)	0.66	9 (18.0%)	6 (24.0%)	0.76
Anastomotic						
Overall	40 (14.9%)	4 (16.0%)	1.00	6 (12.0%)	4 (16.0%)	0.90
Symptomatic	31 (11.5%)	4 (16.0%)	0.73	5 (10.0%)	4 (16.0%)	0.71
Post-transplant cholangiopathy						
Overall	13 (4.8%)	2 (8.0%)	0.83	4 (8.0%)	2 (8.0%)	1.00
Symptomatic	6 (2.2%)	0 (0%)	0.99	2 (4%)	0 (0%)	0.80

Data are expressed as mean (standard deviation) or number (percentage). *p-value referring to Mann-Whitney, Chi-square or Fisher’s test, as appropriate. Abbreviations: SCS, static cold storage; HOPE, hypothermic oxygenated machine perfusion; ALT, alanine aminotransferase; AST, aspartate aminotransferase; LT, liver transplantation.

**Table 3:** Results of logistic and linear regressions of the HOPE effect in the matched cohort.

	Effect*	95% CI	p
Stage 2–3 acute kidney injury	0.29	0.08–0.86	0.024
Severe post-reperfusion syndrome	0.24	0.02–1.11	0.069
Early allograft dysfunction	0.93	0.33–2.50	0.89
ALT peak (IU/L)	−25	−326–+276	0.87
AST peak (IU/L)	−73	−699–554	0.82
Lactate at the end of LT	0.05	−0.68–+0.68	0.89
Grade ≥3 complications	0.91	0.16–5.06	0.87
Acute rejection	2.39	1.62–3.53	0.20
Hospital stay (days)	−2.3	−9.1–+4.4	0.49
ITU stay (days)	0.13	−1.5–+1.8	0.87
Biliary complications	1.26	0.32–4.95	0.70
Anastomotic			
Overall	1.24	0.28–5.49	0.76
Symptomatic	1.52	0.51–4.57	0.56
Post-transplant cholangiopathy			
Overall	0.89	0.15–5.19	0.90
Symptomatic	0.34	0.002–4.41	0.45

*For dichotomous outcomes (acute kidney injury, post-reperfusion syndrome, early allograft dysfunction and biliary complications) effect represents the odds ratio of the outcome in patients treated with HOPE; for continuous outcomes (ALT and AST peak) effect represents the mean variation of the outcome in treated patients. Abbreviations: HOPE, hypothermic oxygenated machine perfusion; CI, confidence interval; ALT, alanine aminotransferase; AST, aspartate aminotransferase.

To obtain more robust estimates of the effect of HOPE on analyzed outcomes, a further analysis using propensity score-based Bayesian model averaging was carried out, which confirmed the association between HOPE use and a 71% and 79% reduction of stage 2–3 AKI and severe PRS, respectively (Table 4). Together with D-MELD, HOPE use was the variable with the highest percentage of inclusion among predictive models of significant PRS. Interestingly, HOPE use was also associated with a reduction of post-LT AST (PI: 13%; effect: −437 IU/L) and ALT peak levels (PI: 23%; effect: −394 IU/L), and of EAD rate (PI: 6%; OR: 0.44).

**Table 4:** Bayesian model averaging models for early post-LT outcomes.

Stage 2–3 Acute kidney injury	Percentage of inclusion	Effect	95% Credible Interval		Probability of significant association btw HOPE and outcome
HOPE	36	0.29	0.09	0.98	98%
Creatinine (mg/dl)	89	1.52	1.09	2.13	
GRBWR	84	0.41	0.22	0.78	
D-MELD	49	1.00	1.00	1.00	
MELD	48	1.06	1.00	1.13	
Donor Age	38	0.98	0.96	0.99	
DRI	6	0.57	0.28	1.17	
BAR	5	1.04	0.99	1.10	
**Severe post-reperfusion syndrome**					
HOPE	16	0.21	0.03	1.66	93%
D-MELD	16	1.00	1.00	1.00	
Graft weight (gr)	13	1.00	1.00	1.00	
Creatinine (mg/dl)	11	1.26	0.99	1.62	
MELD	8	1.04	1.00	1.08	
**Early allograft dysfunction**					
HOPE	6	0.44	0.15	1.31	93%
Macrosteatosis ≥15%	100	8.37	3.74	18.71	
Cold ischemia time (min)	100	1.01	1.00	1.01	
Graft_weight (gr)	8	1.00	1.00	1.00	
GRBWR	8	1.60	0.89	2.87	
**ALT peak**					
HOPE	23	−394.83	−759.48	−30.18	98%
Macrosteatosis ≥15%	100	704.07	419.51	988.63	
Ascites at LT	96	−324.37	−532.67	−116.08	
Cold ischemia time (min)	82	1.71	0.49	2.94	
Donor age	49	−7.22	−12.77	−1.66	
MELD	45	32.12	−1.10	65.34	
Life support	29	−943.02	−1758.08	−127.96	
D-MELD	23	−0.40	−0.87	0.07	
BMI	12	24.93	−2.22	52.07	
BAR	8	−18.17	−40.01	3.67	
**AST peak**					
HOPE	13	−437.76	−933.39	57.87	96%
Macrosteatosis ≥15%	100	1399.31	1011.23	1787.38	
Cold ischemia time	100	3.27	1.68	4.87	
BMI	99	72.65	34.29	111.01	
GRBWR	99	787.44	497.60	1077.29	
Ascites at LT	57	−339.98	−611.01	−68.96	
Donor age	15	−6.94	−14.45	0.58	

The strength of the association between each variable and the analyzed outcome is given by the percentage of inclusion the different models. Only variables with a percentage of inclusion >5% are shown. In case of dichotomous outcomes (acute kidney injury, post-reperfusion syndrome and early allograft dysfunction) effect represents the odds ratio of the outcome for unit variation of the associated variable; for continuous outcomes (ALT and AST peak) effect represents the mean variation of the outcome for unit variation of the associated variable. Upper and lower columns represent the lower and higher values of the likelihood interval. HOPE was associated with a 71%, 79% and 56% mean reduction of the risk of stage 2–3 acute kidney injury, post-reperfusion syndrome and early allograft dysfunction, respectively, and with a 394 U/L and 437 U/L mean reduction of ALT and AST peak, respectively. The probability of significant association (last column) is a measure of the strength of the association and represents the probability HOPE use reduces the risk (i.e. the odds ratio is <1) for stage 2–3 acute kidney injury, post-reperfusion syndrome and early allograft dysfunction, respectively, and the probability HOPE use reduces ALT and AST peak. Abbreviations: LT, liver transplant; GRBWR, graft-to-recipient body weight ratio; MELD, model for end-stage liver disease score; D-MELD, donor age * MELD score; HOPE, hypothermic oxygenated perfusion; DRI, donor risk index; BAR, balance of risk score.

### HOPE effect on biliary complications

After a minimal follow-up of six months, there were 49 (18.2%) and 6 (24%) patients who presented with a biliary complication in SCS and HOPE group, respectively (p = 0.659) (Table 2). In the SCS group, 36 (13.4%), 9 (3.35%) and 4 (1.49%) patients developed an anastomotic complication, post-transplant cholangiopathy, or both, respectively; in HOPE group, 4 (16%) and 2 (8%) patients developed an anastomotic complication or post-transplant cholangiopathy, respectively.

In the whole cohort, there were no differences between groups concerning anastomotic complications (14.9% versus 16%; p = 1) or post-transplant cholangiopathy rate (4.8% versus 8%; p = 0.831). The difference remained not significant even when only symptomatic cases were considered. Incidence of symptomatic post-transplant cholangiopathy was 2.2% and 0% in SCS and the HOPE group, respectively (p = 0.99) (Table 2).

In the matched cohort, there were no differences in terms of the incidence of overall anastomotic complications (12% versus 16%; p = 0.904), symptomatic anastomotic complications (10% versus 16%; p = 0.706), post-transplant cholangiopathy (8% versus 8%; p = 1) or symptomatic post-transplant cholangiopathy (4% versus 0%; p = 0.8) (Table 2). At logistic regression, HOPE use did not have a significant effect on any type of biliary complication (Table 3).

Bayesian model averaging analysis confirmed that HOPE use was not associated with the development of overall, anastomotic or non-anastomotic biliary complications (Table 5).

**Table 5:** Bayesian model averaging models for biliary complications.

	Percentage of inclusion	OR	Lower	Upper
**Biliary complications**				
Graft weight (gr)	95	1.00	1.00	1.00
GRBWR	94	4.94	2.02	12.06
D-MELD	60	1.00	1.00	1.00
Donor age (y)	24	1.02	1.00	1.04
Creatinine at LT (mg/dl)	8	1.28	0.94	1.75
BMI	7	0.89	0.77	1.02
MELD	7	1.00	0.90	1.11
RRT at LT	7	2.94	0.64	13.57
**Anastomotic biliary complications**				
BMI	95	0.81	0.72	0.91
Donor age (y)	45	1.03	1.00	1.05
D-MELD	43	1.00	1.00	1.00
RRT at LT	18	4.80	1.04	22.25
Graft weight (gr)	12	1.00	1.00	1.00
GRBWR	8	2.73	0.39	19.17
MELD	6	0.98	0.85	1.13
Recipient age (y)	6	1.04	0.98	1.09
Creatinine at LT (mg/dl)	6	1.30	0.94	1.81
**Post-transplant cholangiopathy**				
Recipient age (y)	22	0.95	0.90	1.00
Sex (male)	20	0.32	0.10	0.99
Donor BMI	8	0.90	0.77	1.04
BAR	6	0.95	0.87	1.03
RRT at LT	5	0.00	0.00	Inf
Cold ischemia time (min)	5	1.00	1.00	1.01

The strength of the association between each variable and the analyzed outcome is given by the percentage of inclusion the different models. Only variables with a percentage of inclusion >5% are shown. Abbreviations: OR, odds ratio; GRBWR, graft-to-recipient body weight ratio; MELD, model for end-stage liver disease score; D-MELD, donor age * MELD score; LT, liver transplant; BMI, body mass index; RRT, renal replacement therapy; BAR, balance of risk score.

### Patient and graft survival

**Figure 3:**
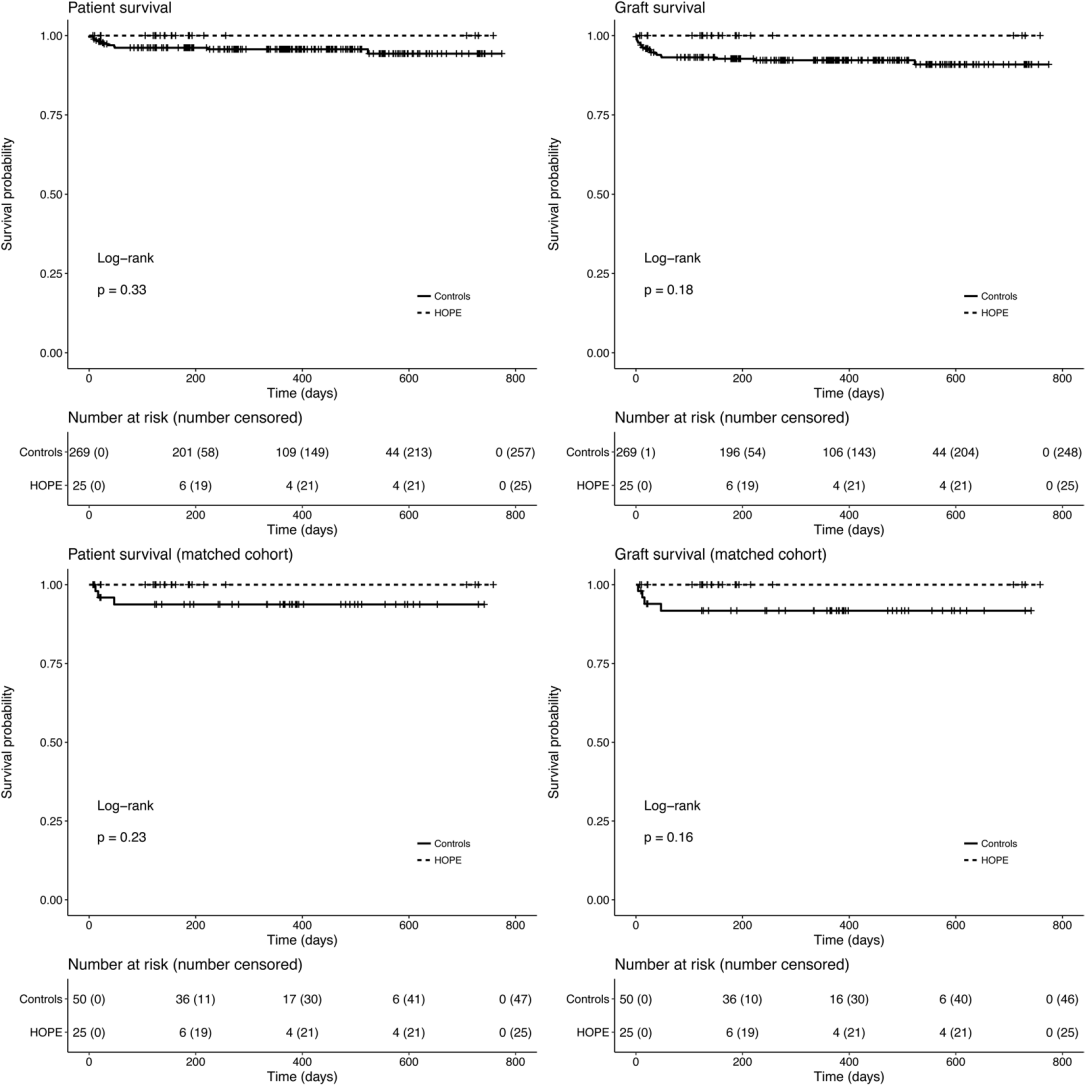
Patient and graft survival curves in the whole cohort and in the matched subset.

Median (IQR) follow-up was 367 (196–525) and 264 (215–288) days in controls and the HOPE group, respectively. In both groups, median survival time was not reached at the end of follow-up. There were no significant differences between HOPE and SCS groups concerning patient and graft survival in both the unmatched and matched cohorts; however, 100% patient and graft survival was observed in the HOPE group (Fig. 3).

### Association between vascular resistance during HOPE and early outcomes

**Figure 4:**
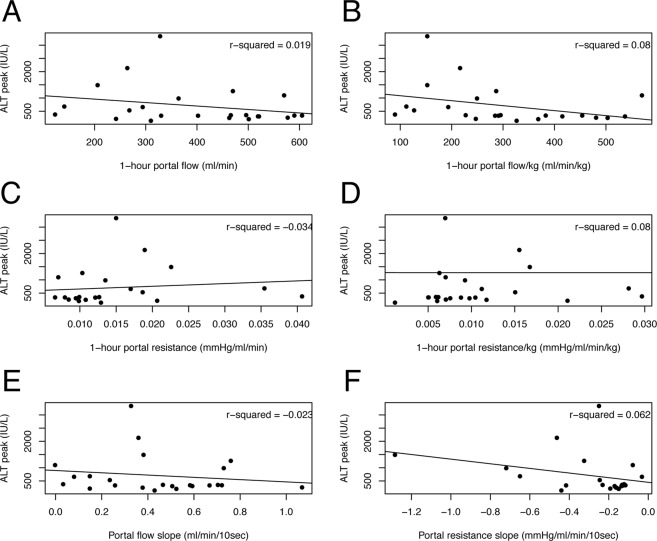
Scatterplots with regression lines showing the association between ALT peak after liver transplantation and portal vein perfusion values: 1-hour flow (**A**), 1-hour flow per kg of graft weight (**B**), 1-hour resistance (**C**), 1-hour resistance per kg of graft weight (**D**), flow slope (**E**) and resistance slope (**F**). ALT, alanine aminotransferase.

**Figure 5:**
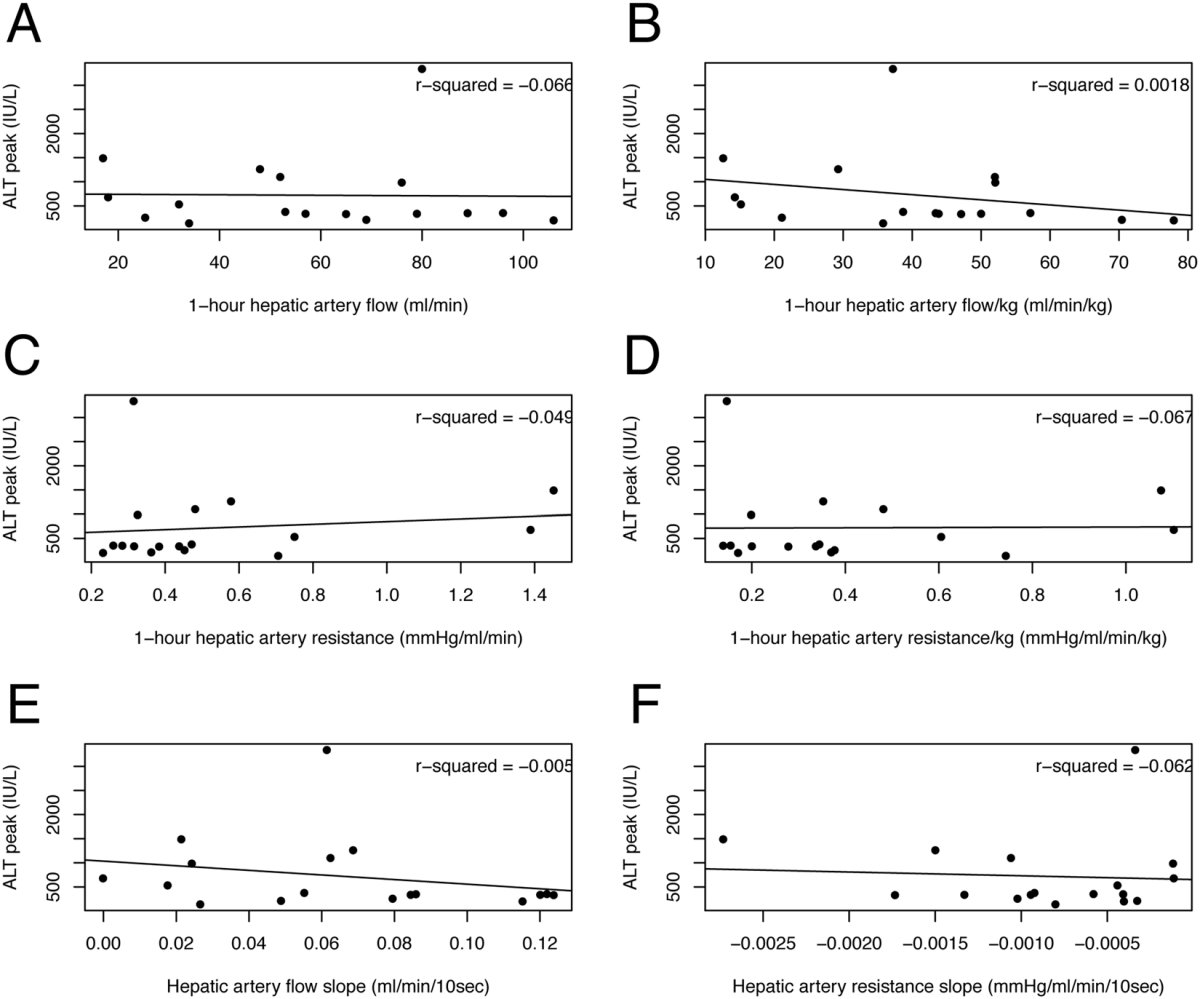
Scatterplots with regression lines showing the association between ALT peak after liver transplantation and hepatic artery perfusion values: 1-hour flow (**A**), 1-hour flow per kg of graft weight (**B**), 1-hour resistance (**C**), 1-hour resistance per kg of graft weight (**D**), flow slope (**E**) and resistance slope (**F**). ALT, alanine aminotransferase.

Table 6 summarizes perfusion parameters measured during or at the end of the first HOPE hour according to the development of EAD. Patients who did not develop EAD after LT showed a steeper decrease of hepatic artery resistance throughout perfusion (68 versus 48 mmHg/ml/min/10 sec*10^5^; p = 0.035). Flow and resistance values showed poor correlation with post-LT ALT peak (Figs 4 and 5) and AST peak (data not shown).

**Table 6:** Hepatic artery and portal vein perfusion values according to the development of early allograft dysfunction after LT.

	Portal vein circuit			Hepatic artery circuit		
	No EAD (n = 17)	EAD (n = 8)	p	No EAD (n = 17)	EAD (n = 8)	p
1h-Flow (ml/min)	392	396	0.95	61	50	0.51
1h-Flow/kg (ml/min/kg)	303	299	0.95	44	30	0.22
1h-resistance (mmHg/ml/min)	0.02	0.01	0.73	0.49	0.70	0.34
1h-resistance/kg (mmHg/ml/min/kg)	0.01	0.01	0.74	0.40	0.48	0.66
Flow slope (ml/min/10 sec)	0.41	0.51	0.47	0.07	0.06	0.71
Resistance slope (mmHg/ml/min/10 sec*10^5^)	−2	−4	0.16	−68	−48	0.03

Flow and resistance values were collected at the end of the first hour of machine perfusion. Slopes were calculated as the mean variation per 10-seconds interval. Abbreviations: LT, liver transplant; EAD, early allograft dysfunction.

## Discussion

In this paper, we show that HOPE use in grafts from DBD donors was associated with a significant reduction of stage 2–3 AKI and severe PRS rate. Moreover, HOPE use was associated with a lower incidence of EAD and reduced post-LT AST and ALT peak. In our experience, HOPE did not reduce the length of hospital or ITU stay, acute rejection rate, and grade ≥3 surgical complications rate. In particular, HOPE did not seem to affect the incidence of biliary complications. Although there were no significant differences in patient and graft survival, recipients of HOPE-treated grafts had 100% patient and graft survival.

In last years, the necessity to cope with the detrimental effects of ischemia-reperfusion injury on grafts from extended criteria donors prompted a renewed interest towards machine perfusion techniques. The basics of ischemia-reperfusion injury, as applied to different organs and settings, have recently been reappraised. The first event in the induction of pathological ischemia-reperfusion injury appears to be a burst of mitochondria-derived superoxide (O_2_^−^), leading to the production of other reactive oxygen species, like hydrogen peroxide (H_2_O_2_). Reverse electron transfer through mitochondrial complex I is the main mechanism of superoxide production as a result of the high protonmotive force that is created upon reperfusion^34,35^. This leads to aspecific inflammation, Kupffer cells activation, dendritic cells maturation, endothelial expression of adhesion molecules, neutrophils infiltration, platelet aggregation and, finally, impairment of the microcirculation^36^. In the experimental setting, HOPE has shown to improve liver graft preservation as compared to SCS through continuous shear stress over sinusoidal endothelial cells, washout of metabolites produced during ischemia, increase of cellular adenosine triphosphate content, better peribiliary vascular plexus perfusion, and decreased mitochondrial release of reactive oxygen species at reperfusion by reverse electron transfer through mitochondrial complex I^19,34,37–43^. These effects would be particularly beneficial in steatotic grafts, which suffer from a baseline impairment of microcirculation and are therefore particularly vulnerable to the decreased synthesis of protective molecules (nitric oxide, thrombomodulin) determined by the lack of mechanical shear stress over the endothelium during SCS^44^. Clinical data concerning HOPE use are still limited and mainly focused on grafts from DCD donors. In their pioneering papers, Guarrera *et al*.^9,10^, using hypothermic machine perfusion (HMP) without active oxygenation (O_2_ pressure was around 230 mmHg due to passive oxygenation), observed lower transaminases, bilirubin and creatinine peak after HMP, and no cases of post-transplant cholangiopathy. The Zurich group found that 1–2-hours end-ischemic HOPE in DCD LT is associated with lower transaminases peak, better synthetic function, lower EAD and post-transplant cholangiopathy rate, and better patient and graft survival^7,8,45^. From the same group, Kron *et al*.^11^ published a study in which six recipients of HOPE-treated steatotic livers (20–40% macrovesicular steatosis), 5 of which from DCD donors, were compared to 12 recipients of steatotic grafts (20–60% macrovesicular steatosis) from DBD donors, matched for donor and recipient age and total preservation time. HOPE was associated with lower AST peak, lower requirement of renal replacement therapy, shorter intensive care unit stay and better 1-year patient survival. The Groningen group has observed that end-ischemic dual-HOPE of grafts from DCD donors is associated with reduced biliary injury and better preservation of peri-biliary glands compared to SCS, and with a lower incidence of post-transplant cholangiopathy^46,47^. In Italy, combined use of normothermic regional perfusion^48,49^ and HOPE has allowed for the successful implementation of DCD LT despite the 20-minute no-touch period imposed by Italian law^50^.

In our experience, the most evident benefit of HOPE use was an improved hemodynamic stability at graft reperfusion in the recipient and a lower incidence of moderate-to-severe AKI after transplant. These two findings are likely closely interlinked. Improved hemodynamic stability upon graft reperfusion is consistently observed when dynamic preservation techniques – both normothermic and hypothermic machine perfusion - are employed and could be related to the reduction of ischemia-reperfusion injury, the washout of metabolites produced during ischemia and to the “vascular bed recruitment” effect of machine perfusion^17,45^. Recently, a paper from the Groningen group shed further insight on this topic by showing that HOPE-preserved liver grafts, as opposed to what is commonly observed after SCS, release potassium in the perfusate during machine perfusion and take up potassium upon reperfusion, preventing the development of the acute hyperkalemia that is frequently associated with severe PRS^51^. Severe post-reperfusion syndrome has been shown to be a marker of more severe ischemia-reperfusion injury and also to be associated with an increased rate of AKI after LT^52^.

The etiology of AKI after LT is multifactorial, including pre-LT renal function impairment, hemodynamic instability during LT operation, factors related to surgical technique, drug toxicity and ischemia-reperfusion injury. To this regard, the strong relationship between ischemia-reperfusion injury and AKI is supported by the association between post-LT AST peak and AKI^53,54^. Our results suggest that the lower stage 2–3 AKI rate in the HOPE group could be related to better hemodynamic stability during LT and could arguably be considered as a marker of reduced ischemia-reperfusion injury. It is likely that our study was underpowered to detect a significant difference in post-LT dialysis, given its low incidence.

Our findings were less compelling concerning the reduction of EAD rate and transaminases peak in the HOPE group, which appeared to be mainly related to other factors such as macrovesicular graft steatosis, graft weight and cold ischemia time. Furthermore, although we observed a 100% patient and graft survival in the HOPE group, we were unable to demonstrate a significant advantage in terms of patient and graft survival. This lack of significance could be due to the limited sample size. Another potential confounder is represented by the longer preservation time in HOPE-treated patients. Based on the available experimental evidence^30^, we scheduled backtable graft preparation and recipient operation in order to allow a minimum perfusion time of 90 minutes. Although we and others have also used HOPE in cases where, due to logistic issues, a longer ischemia time is expected^55^, it is unclear if HOPE can be considered a way to prolong preservation time. We expect that, as we continue to gain confidence in the device set-up and in theatre time organization in HOPE cases, this difference will become less evident in the near future.

Unexpectedly, we did not observe any effect of HOPE treatment on the incidence of biliary complications, and in particular of post-transplant cholangiopathy. However, two considerations must be made. Firstly, the clinical evidence of a favorable effect of HOPE on the incidence of biliary complications is derived from DCD LT^7,45–47^, which is burdened by a higher rate of biliary complications and it is consequently a more suitable setting to highlight the beneficial effect of any type of intervention. Two recent studies on normothermic machine perfusion^13,56^ highlighted the difficulty in demonstrating a significant effect on this clinical outcome. Secondly, the vast majority of our patients had a T-tube cholangiogram performed three months after LT, which also made it possible to detect asymptomatic biliary abnormalities. These usually go undetected unless imaging studies are systematically obtained. Notably, incidence of symptomatic post-transplant cholangiopathy was 0% in the HOPE group. It could be argued that a six-month follow-up was insufficient to detect biliary complications presenting later after LT, limiting the value of our results. However, most ischemia-reperfusion injury-related biliary complications, and especially post-transplant cholangiopathy, present within this time frame^57^. Thus, it is unlikely that a longer follow-up would have substantially changed our results.

In this study, we also sought to explore HOPE’s potential as tool for graft quality assessment. At present, the inability to assess graft quality during hypothermic machine perfusion is one major limitation of the technique. Therefore, we investigated whether the value of vascular graft resistance and flow patterns during the first hour of machine perfusion can be used as predictors of ischemia-reperfusion injury and postoperative graft function. In kidney transplantation, higher vascular renal resistance at the end of hypothermic machine perfusion is a risk factor for delayed graft function and 1-year graft loss, but its predictive value is low^58,59^. In this study, we observed poor correlation between resistance values and patterns, and post-LT transaminases peak (Figs 4 and 5). However, patients who did not develop EAD had a significantly steeper decline in arterial graft resistance during the first hour of machine perfusion, which suggest that this parameter may potentially play a role in graft quality assessment. Obviously, this finding must be interpreted with caution and the value of arterial resistance slope should be weighed against other well-known predictors of post-LT graft function. Hopefully, ongoing studies on perfusate analysis will increase HOPE potential as a tool for graft quality assessment (Dutkowski P. personal communication at 8^th^ International Meeting on Transplantation from DCD; September 13–14, 2018; Milan, Italy).

This study represents the retrospective evaluation of a pilot experience at a single center and it has therefore many limitations, including its retrospective nature, limited numerosity and arguably a lack of power to achieve statistical significance for many analyzed outcomes, in particular patient and graft survival. The main limitation is certainly represented by the criteria that were used to consider HOPE use. As donor age is the main risk factor for biliary complications^60,61^, whereas graft steatosis is a risk factor for EAD and early graft failure^62,63^, we chose to employ HOPE in case of advanced donor age or graft steatosis, using donor BMI and direct graft inspection as surrogates of pre-transplant histologic graft assessment. This was dictated by the difficulty of obtaining routine pre-LT evaluation of graft biopsies in our setting. Unfortunately, donor BMI and graft evaluation by the retrieval surgeon had a poor predictive value for graft steatosis, resulting in only 5 out of 11 grafts identified as steatotic actually harboring a macrovesicular steatosis ≥15%. This led to the selection of a rather heterogeneous donor population mainly characterized by older age (74.3 versus 60.9 years; p < 0.001) and, consequently, by higher DRI (2.09 versus 1.78; p = 0.004), but with a comparable rate of macrosteatosis ≥15% (16% versus 12.6%; p = 0.54) as compared to the reference group. We acknowledge that these features would hardly be defined as “extended criteria” in present days. It should be noted, however, that this limitation is shared by many published studies in which different machine perfusion techniques have been applied to discarded livers, which are far from being a homogeneous population^10,15–17^.

Although our results can be considered promising, the lack of a clear advantage in terms of patient and graft survival and biliary complications is questioning. Apart from the limited sample size, it is likely that our use of HOPE in a rather heterogeneous and low-risk setting hampered our ability to show a major benefit of the technique. This highlights the necessity to identify the areas in which application of machine perfusion techniques would yield a significant clinical advantage over static cold storage. As recently proposed, future studies will have to focus on relevant clinical endpoints and include a health economics analysis^64^. At present, there are several ongoing randomized controlled trials (source: https://clinicaltrials.gov) evaluating the clinical impact of HOPE in different settings, which will hopefully help drawing the line beyond which HOPE use is associated with a relevant and economically sustainable clinical benefit.

In conclusion, in our experience end-ischemic HOPE in DBD LT was associated with a significant reduction of severe PRS, stage 2–3 AKI rate and EAD, and with a lower transaminases peak. These findings suggest a reduction of ischemia-reperfusion injury in HOPE-treated grafts. The slope of arterial graft resistance during HOPE may represent an indicator of graft quality, to be integrated with other prognostic factors. This study represents the report and analysis of a real-life attempt at improving the outcome of our patients and, given the scarcity of data in this context, we believe it could be of interest to other Centers that are planning to adopt this technology.

## Data Availability

All relevant data are reported in the paper. Further supporting data will be provided upon written request to the corresponding author.
